# Qualitative-archival research on migration is not just preservation

**DOI:** 10.12688/openreseurope.17804.2

**Published:** 2025-08-29

**Authors:** Federica Manzoli, Matteo Al Kalak, Maria Chiara Rioli

**Affiliations:** 1 Department of Studies on Languages and Culture, University of Modena and Reggio Emilia, Modena, Emilia Romagna, 41121, Italy

**Keywords:** Migration, Migration policies, Qualitative research, Research-policy relations, Social Innovation

## Abstract

Qualitative-archival research is not just “preservation”: it shows the potential of providing dynamic and interlinked information, including legal frameworks, policy documents, historical context, and original narratives, capable of supporting policies in the long term. Besides this, it is a potential tool to foster the agency of migrants.

Morover, qualitative-archival research can be a powerful tool for social innovation, as conducting qualitative research, based on individual and collective narratives, is a necessary basis to non-emergency policies and to social work planning and evaluation. It helps to explain the reasons behind the migration phenomena and to contextualise them where it is most needed, whether in political, educational, labour, health or reception settings.

*Space, time and people* are the three main enablers of better qualitative and archival research-policy relations: qualitative archival research offers contextual and comparative tools to investigate in-depth local features and to possibly extend them to the national and international levels.

These results emerge from a consultation with more than 150 stakeholders among researchers, policymakers, practitioners, migrant associations, governments, civil society/non-governmental organisations and migrants in seven countries around the Mediterranean, in the frame of the H2020 project ITHACA - Interconnected Histories and Archives for Migrant Agency.

## Disclaimer

The views expressed in this article are those of the author(s). Publication in Open Research Europe does not imply endorsement of the European Commission.

## Introduction

This Open Letter concentrates on qualitative archival research as this is the main focus of the EU-funded H2020 ITHACA – Interconnected Histories and Archives for Migrant Agency - project. From January to June 2023, eight of the eleven partners ^1^ organised a series of Policy Council Events (PCEs) with the aim to enable a broad group of stakeholders to meet and exchange thoughts, views and proposals at local, national and international levels on the topic of the relationship between qualitative-archival research and policies.

Around 150 policymakers, researchers (52) in migration studies, sociologists, anthropologists, historians, NOGs/migrant association representatives and migrants (50), communicators/journalists (5), intergovernmental organisation representatives (5), practitioners (11), university students (13) and lawyers (5) participated, gathering in 12 meetings in 7 of the ITHACA countries: Italy (Rome, Milan, Modena), Greece (Athens), the Netherlands (Leiden), Morocco (Rabat, Ifrane), Tunisia (Tunis, Tataouine), Azerbaijan (Baku, Ahan Village) and Jordan (Amman). 

After building a map of the interested/affected persons, the project partners recruited the participants and facilitated the meetings. The main criterion to contact and invite the participant was based on the categories set by the Horizon2020 topic “MIGRATION-09-2020 - Narratives on migration and its impact: past and present”, which identified the main stakeholders among “policy-makers, civil society organisations, citizens and migrants”. The resulting stakeholder map took into consideration the need to balance different points of view, reflected in the mix of different stakeholders participating, including a high percentage of migrants/migrant associations and NGOs (about 1/3), researchers, practitioners, and policy-makers.

A common guideline was shared to make the results comparable. The guidelines were based on three main topics, corresponding to three main key findings here reported: the first is related to the main advantages taken by QAR in supporting migration policies, the second is related to the obstacles in this type of research-policies relations, and the third is related to the factors that can facilitate more effective collaboration between the two. The open letter closes with a series of recommendations dedicated to the same groups that contributed to the discussion on these issues.

### Qualitative-archival research in the migration field

Qualitative-archival research (QAR) is based on discourse. It collects data in the form of individual or collective interviews, audio, written or visual texts (video, images or multimedia), thereby “interpreting” social contexts ( Bauer & Gaskell, 2000; Erdal *et al.*, 2022).

QAR helps to build policies that are not based on abstract assumptions but are instead based on individual and collective experiences ( LERU, 2009; Rodari *et al*., 2012), as qualitative research methods, such as interviews, focus groups, and self-narratives, offer a nuanced understanding of migration practices, motivations, and challenges ( Fouskas, 2016; Scholten, 2018).

Analysing in-depth stories, experiences and testimonies from the different actors involved in the migration discourse, migrants, policymakers, and practitioners working in the field, as well as from those who design and evaluate migration policies, QAR facilitates an exploration of the values and reasons behind migration in the different cultures and can assist in explaining the different elements of migration of varying significance to the overall concept ( Reed & Rudman, 2023; Theodoros, 2016).

As well, while assisting the understanding of the experience of migration - the journey, the arrival, the practical implications, the risks faced by migrants – QAR deepens how this perspective affects decisions, also identifying how countries, politicians and administrations respond when adopting specific reception procedures.

When using QAR results, however, users should always adopt a critical approach. Written, audio, video, images or multimedia may be conceived and interpreted differently in different cultures. This is the reason why, regardless of the type of archived qualitative data that can be conceived and interpreted differently in different cultures, before use, users must be aware of *how* they were collected and stored ( Stewart & Shaffer, 2021; Valles *et al*., 2011). In this respect, the presence of metadata is an effective indicator for users of databases, repositories, and archives since their availability is a sign of high quality research.

## Key-Finding 1: Potentials of using Qualitative-Archival Research to link migration research and policies

Uses of QAR are in the assessment of migration policies, for their improvement and for guaranteeing consistency with other relevant public policies.

*Being able to access qualitative research data makes it possible to implement interventions that are more fitting for the territory in which we live, going beyond the prejudices that, even with the best intentions, most of us hold. For example, if I find that I am managing the reception of a migrant, I ask myself about his or her characteristics, his or her specific features, the consequences for him or her of not having a home.* (Practitioner, NGO, Modena, Italy)

Faraway from being just a tool for preservation, the findings of QAR are a social innovation tool, providing an opportunity to:

- Discuss stereotypes by exploring the multifaceted experiences of migrants;
- Pose new questions to policy-decision makers and practitioners, by allowing them to ground on in-depth knowledge, based on a research process and evidence-based results;
- Monitor and evaluate existing policies, by using methods that allow an extensive understanding of contexts;
- Improve and create new policies by exploring stories, perceptions, needs and attitudes of migrants coming from different cultures, commonalities and differences;
- Base decisions on the complexity of the phenomenon and not on emergencies, by using methods that allow an in-depth understanding of contexts and individual/group narratives;
- Save money, by avoiding starting from scratch (situations and solutions have often already been studied and policy decisions can take advantage of existing experiences);
- Network with the stakeholders involved, by sharing stories and suggestions with other practitioners, researchers and policymakers, and by facilitating access to migrant communities.

A further crucial point discussed by ITHACA stakeholders was *ethics*. Terms such as “transparency”, “respect”, “independence”, “ethics”, and “honesty” frequently appear in their arguments when discussing *how to use* QAR. When asking for fair research based on a contextualised, interrelational, and reflexive methodology that can complement and interpret quantitative data, not just researchers but also migrants, associations representing them and practitioners ask for epistemic justice ( Corti, 2011; van Liempt & Bilger, 2018).

The main ethical risk in the relationship between research (all research fields and methods) and policies is instrumentality. Knowledge can be used to substantiate specific political claims or ideas, or to foster populism.

The main opportunity provided by responsible QAR is that it can identify questions and views that help to dismantle stereotypes, with beneficial repercussions not only in terms of ethical issues behind the research and its use, but also in relation to the development of better practical solutions and services.

## Key-Finding 2: Obstacles in research-policy relations

Across the countries involved in ITHACA, the structural obstacles identified by stakeholders which prevent policymakers from using research results include:

- regulatory gaps;
- specific aspects of the local situation;
- administrative practices;
- different timeframes.

*Caught up in emotion, we believe the arrival of migrants is the heart of the matter. The arrival of migrants makes it clear to us that there is a problem. New people arrive. If migrants arrive - be they children or adults - and do not speak the language, or speak it differently, or have different experiences, first of all, we have to understand who these people are. A repository of stories becomes essential for understanding who they are, it is a first step towards useful and manageable decisions*. (Policy maker, Italy)

A further reason for the ineffective relationship between research and policies is *politics*.

Policy planning and decisions vary according to the political agenda of each government, and the research results - especially if they are not aligned with current government programmes - are often not taken into consideration.

From the research side, some practitioners and policymakers call for more policy-oriented research. Their findings need to be challenged, debated and tested before they can provide a reliable basis for any recommendations.

The *structural* and *political* gaps here identified are behind the insufficient utilisation of existing and potential QAR.

## Key-Finding 3: Enhancing qualitative research-policy relations

What factors can facilitate more effective collaboration between research and migration policies?

Three factors emerge.

The first is the “Coverage”: migration is a vast and multidimensional issue which varies according to the political, economic, cultural, and societal conditions of the host and origin countries, the individual motivations to migrate and the meaning given by governments to the freedom of movement involved in migration flows. The use of QAR in migration policies crucially depends on the geographical dimension in which it operates. The dialogue held by policymakers and practitioners/migrants with researchers in small towns might be more direct and frequent than the dialogue that takes place internationally. However, to design effective policies, QAR offers contextual and comparative tools for thoroughly investigating the local features and extending them to national and international levels, covering the global-local nexus. From the methodological perspective, one example is given by the manner in which ITHACA’s PCEs were conceived and implemented: they started at the local level and expanded their findings to national and international levels. In this way, commonalities and differences emerged and gave rise to practical recommendations.

The second factor is the “Timeline”: in order to enhance the research-policy relationship, there is a need for synchronicity, establishing a common programme from the outset. This would help both researchers and policymakers to gain a clearer understanding of the field and to make more informed decisions.

As migration is a long-term phenomenon, research should be addressed as an integral part of the political life of a city, region and state.

An economist and ex-minister for education in Italy explained:

*If politics is short-term, emotional, incapable of planning, it is also incapable of posing long-term questions to research. Politics is no longer based on values and interests, but on emotional states. Research must study, analyse, communicate migrations, demonstrating that policies should not be decided on the basis of dramatic events or extemporaneous reactions, but that we must look at them from a long-term perspective.*

The third factor is “Togetherness”: when research is to be used as a tool for collaboration, there is a need for research and politics to come together more often and more regularly, to ascertain the common aspects, the development steps and the ultimate goal. A policy maker, who took part of the international meeting that closed the round of the ITHACA policy councils in Ifrane (Morocco), affirmed:

*An effort must be made to reposition politics in line with research, but also with those who perform research. This requires efforts on both sides, a common recognition.*

In this context, the degree of liberty of expression and democratic participation of citizens and civil society organisations differs substantially between European and other MENA countries involved in the policy councils, resulting in the limited possibility of constructing a real dialogue between researchers, migrants, practitioners and policymakers.

The need for platforms to facilitate regular consultations, joint working groups, and knowledge-exchange was mentioned at several meetings.

By establishing open communication channels, stakeholders can benefit from each other's expertise, insights, and perspectives, leading to more informed decision-making, policy formulation and evaluation.

Finally, in QAR, policymakers and academics should always involve migrants and migrant communities in a collaborative effort, ensuring their voices are heard and integrated into policy discussions, services planning and decisions.

*There must be a political wish to listen to stories of migrants. Listen to them directly implies a political responsibility… The logic of power is hardly discussed, making policies disconnected from the reality on the ground” (Researcher, Tunisia)*

## Recommendations for policymakers, practitioners, researchers

QAR can provide invaluable resources for research-based migration policies. The need for reflexivity, contextualisation, understanding of the time-space conjuncture, interdisciplinarity, and critical action are the premises for researchers, policymakers, practitioners and migrants themselves.

In the extensive research-action effort here described

, the ITHACA Policy Councils provided them with the following recommendations:

1. Consider qualitative and archival research for a more substantive and nuanced understanding of the complexities of policy implementation and an opportunity to formulate the “correct” questions in your daily work.
2. Clarify the underlying problem, where it comes from and how it is seen locally, nationally, and internationally, according to your objectives.
3. Understand the (local, national, international) policy-making process in order to provide relevant and timely advice.
4. Identify the power relations in the context in which you study/operate.
5. Before participating in research or using research resources, reflect on your role (policymakers might see research in instrumental terms, while researchers might investigate the intrinsic value of policy).
6. Keep in mind your stakeholder map (including universities, research centres, foundations, CSOs, NGOs, journalists and communicators), in order to exploit the targeted results.
7. Think in an interdisciplinary manner: historians, sociologists, anthropologists, archivists, ICT researchers should work together with migrants, practitioners, policymakers and other interested parties.
8. Declare the analytical perspective you apply when reading through the narrations collected.
9. Use the research results to deconstruct mainstream narratives.
10. Be specific: the more prevalent a specific topic is, the greater the likelihood of piquing the interest of the government or other organisations.
11. Maintain a critical approach, independently from the source.
12. Reflection and self-reflection when using QAR include the language used to acquire and communicate on migration issues.

## Ethics and consent statement

Ethics and consent statement were not required.

## Data Availability

No data are associated with this article.

## Footnotes

^1^ITHACA project partners: University of Modena and Reggio Emilia (IT), Sorbonne Université (FR), Universiteit Leiden (NL), United Nations High Commissioner for Refugees (CH), University of Athens (GR), French Institute of the Near East (CNRS, FR), University of Milan (IT), Archivio delle Memorie Migranti (IT), ARCS Tunisie (TN), Institue of Geography – National Academy of Sciences of Azerbaijan (AZ), Université Al Akhawayn D’Ifrane (MA).
