# Supplementary material for: Qualitative-archival research on migration is not just preservation
Source: Open Res Eur. 2025 Aug 29;4:149. Originally published 2024 Jul 16. [Version 2] doi: 10.12688/openreseurope.17804.2 (PMC12409257; doi:10.12688/openreseurope.17804.2)
Supplement: Peer review history

# Reviewer report: Reviewer response for version 2

**DOI:** 10.21956/openreseurope.21852.r59290

**Published:** 2025-09-12

**Authors:** Dieter Schlenker

**Affiliations:** 1 European University Institute, Fiesole, Toscana, Italy

no further comments

Where applicable, are recommendations and next steps explained clearly for others to follow? (Please consider whether others in the research community would be able to implement guidelines or recommendations and/or constructively engage in the debate)

Yes

Does the article adequately reference differing views and opinions?

Yes

Are all factual statements correct, and are statements and arguments made adequately supported by citations?

Yes

Is the rationale for the Open Letter provided in sufficient detail? (Please consider whether existing challenges in the field are outlined clearly and whether the purpose of the letter is explained)

Yes

Is the Open Letter written in accessible language? (Please consider whether all subject-specific terms, concepts and abbreviations are explained)

Yes

Reviewer Expertise:

Modern and Contemporary History, Archival Science, International Relations, Journalism and Communication

I confirm that I have read this submission and believe that I have an appropriate level of expertise to confirm that it is of an acceptable scientific standard.

# Reviewer report: Reviewer response for version 2

**DOI:** 10.21956/openreseurope.21852.r59291

**Published:** 2025-09-03

**Authors:** Irene Gutiérrez Torres

**Affiliations:** 1 Vrije Universiteit Brussel, Brussels, Belgium

I think the new version has been improved and the article is ready to be indexed. I have no further comments to make.

Where applicable, are recommendations and next steps explained clearly for others to follow? (Please consider whether others in the research community would be able to implement guidelines or recommendations and/or constructively engage in the debate)

Yes

Does the article adequately reference differing views and opinions?

Partly

Are all factual statements correct, and are statements and arguments made adequately supported by citations?

Partly

Is the rationale for the Open Letter provided in sufficient detail? (Please consider whether existing challenges in the field are outlined clearly and whether the purpose of the letter is explained)

Yes

Is the Open Letter written in accessible language? (Please consider whether all subject-specific terms, concepts and abbreviations are explained)

Yes

Reviewer Expertise:

Forced migration, borders, film, participatory research, participatory filmmaking, self-representation

I confirm that I have read this submission and believe that I have an appropriate level of expertise to confirm that it is of an acceptable scientific standard.

# Reviewer report: Reviewer response for version 1

**DOI:** 10.21956/openreseurope.19247.r42243

**Published:** 2024-08-06

**Authors:** Dieter Schlenker

**Affiliations:** 1 European University Institute, Fiesole, Toscana, Italy

The Open Letter is a result of the H2020 project ITHACA: Interconnected Histories and Archives for Migrant Agency. It calls for qualitative archival research to support effective policies and the work of practitioners in the field of migration. In the view of the authors, qualitative archival research provides contextualised information and the necessary knowledge to support the development of long-term policies in the field. The individual and collective narratives of historical processes documented in the archives explain the main political, educational, health, and other reasons behind the migration phenomenon, and help interpreting local events within the broader national and international contexts. The project team organised a series of policy council events to develop a proposal on the advantages, the obstacles and raising of effectiveness in the use of qualitative archival research for policies in the field of migration.

 The rationale for the Open Letter is clearly explained. The authors wish to avoid abstract policy making by making use of the numerous experiences documented in archival sources. These show how, at the given time, societies, politicians and administrations responded to migration. The open letter intends to avoid stereotypes and to gain instead knowledge from past experiences for the drafting, managing, as well as monitoring and evaluating of policies. QAR should help understanding the complexities of migration as a phenomenon and not as single event. The letter also names the obstacles in the relation between policy development and research, which consist in regulatory gaps, local specificities, administrative practice and timeframes, as well as political structural constraints and gaps.

 The Open Letter refers to the different point of views of research on the one side and policy makers on the other. Key finding 3 explains that the different views and opinions on migration are based on the difficulty to capture the numerous and multidimensional issues of migration in the specific political, economic, cultural and societal context. Moreover, research and policy may not be synchronic in their relationship, while research should be considered integral part of policy making. Finally, a lack of cooperation consists between research, while QAR can help building a constructive dialogue between societies in the field of migration.

 The factual statements on which the open letter is based, are supported by various citations, quotes, references and notes. As collaborative output of a consultation process with 150 stakeholders in seven countries that participated in the ITHACA project, the letter editors acknowledge the numerous authors.

 The letter is addressing a transnational and multilingual audience, comprising researchers in migration studies, sociologists, anthropologists, historians, migrant association representatives, communicators, intergovernmental organisation representatives and lawyers. The 150 stakeholders involved in the drafting were based in seven countries: Italy, Greece, the Netherlands, Morocco, Tunisia, Azerbaijan and Jordan. The letter maintains a readable and accessible language and style.

 The Open Letter ends with a set of 11 recommendations for policy makers, practitioners and researchers to consider qualitative archival research for a better understanding of the complexities of the phenomenon of migration. The recommendations are clearly formulated and summarise the key findings of the ITHACA research project.

Where applicable, are recommendations and next steps explained clearly for others to follow? (Please consider whether others in the research community would be able to implement guidelines or recommendations and/or constructively engage in the debate)

Yes

Does the article adequately reference differing views and opinions?

Yes

Are all factual statements correct, and are statements and arguments made adequately supported by citations?

Yes

Is the rationale for the Open Letter provided in sufficient detail? (Please consider whether existing challenges in the field are outlined clearly and whether the purpose of the letter is explained)

Yes

Is the Open Letter written in accessible language? (Please consider whether all subject-specific terms, concepts and abbreviations are explained)

Yes

Reviewer Expertise:

Modern and Contemporary History, Archival Science, International Relations, Journalism and Communication

I confirm that I have read this submission and believe that I have an appropriate level of expertise to confirm that it is of an acceptable scientific standard.

# Reviewer report: Reviewer response for version 1

**DOI:** 10.21956/openreseurope.19247.r42249

**Published:** 2024-07-26

**Authors:** Irene Gutiérrez Torres

**Affiliations:** 1 Vrije Universiteit Brussel, Brussels, Belgium

**Overall comments**

 The Open Letter explores some of the potentials and challenges of using qualitative-archival research in migration to support an effective research-policies link. By using QAR, researchers and practitioners working in the field can 1) provide dynamic and interlinked information among researchers, practitioners, policymakers, governments, non-governmental organisations, and migrants; 2) incorporate legal frameworks, policy documents, historical contexts and original narratives to inform policy in the long term; and 3) promote the agency of migrants.

 The research is part of the EU-funded H2020 project ITHACA - Interconnected Histories and Archives for Migrant Agency. The methodology is based on qualitative archival research. The process consisted of building a stakeholder map to guide the recruitment phase carried out by the project partners and facilitating 12 meetings in 7 countries where 150 participants shared perspectives on the relationship between qualitative-archival research and migration policies at local, national and international levels.

 The data collection took place from January to June 2023. Through comparative analysis, the authors addressed three main topics: 1) the advantages of using QAR in supporting migration policies based on lived experiences instead of abstract assumptions; 2) the challenges of using QRA concerning the migration research-policies nexus; and 3) recommendations for establishing a more effective collaboration between migration research and policies through the use of QRA.

**Abstract**

 Acknowledging that the text format is an Open Letter, it would be more convenient to present a traditional abstract to gain coherence because now the body of the text does not have the same tone as the abstract. A solution could be that the abstract is formulated in the traditional form. At the same time, the body of text, especially the findings, adopts the tone of the current abstract in the form of an open Letter as a sequence of findings (advantages and challenges) and recommendations.

**Methodology**

 The methodology mentions more than 150 stakeholders and that the ITHACA partners organised the twelve meetings in seven countries: Italy, Greece, the Netherlands, Morocco, Tunisia, Azerbaijan, and Jordan. However, there are recruitment decisions that would need further explanation. For example, which ITHACA partners are in the seven countries? How did they recruit the participants, under which criteria? How many were researchers, policymakers, and governments, and how many were practitioners, migrant associations, civil society/nongovernmental organisations, and migrants? Is there any rationale in the recruitment to balance visions on migration from below and from above? Since qualitative-archival research is based on discourse analysis, and institutional discourses differ from civil society and migrant discourses, such balance (or imbalance) in the recruitment phase is crucial for the analysis section.

**Theoretical framework**

 The paper is an Open Letter, so the theoretical section is shorter than usual. A possible solution might be introducing the four first paragraphs of the key finding-1 section in the introduction. Perhaps this section could start directly in paragraph five: “Uses of QAR…” and be named, for example, as *Potentials of using Qualitative-archival Research to link migration research and policies*.

 Secondly, it would be beneficial to include a paragraph addressing the ethical aspects of using QRA in research in general. For instance:

 Valles, M. S., Corti, L., Tamboukou, M., & Baer, A. (2011, October). Qualitative archives and biographical research methods. An introduction to the FQS special issue. In *Forum Qualitative Sozialforschung (FQS)* (Vol. 12, No. 3). FQS.

 And in (forced) migration research in particular. Some possible examples:

 Stewart, E., & Shaffer, M. (2021). The ethical and practical challenges of archiving refugee accounts: Reflections from two research projects in the UK. *Bulletin of Sociological Methodology/Bulletin de Méthodologie Sociologique*, *150*(1), 51-69.

 van Liempt, I., Bilger, V. (2018). Methodological and Ethical Dilemmas in Research Among Smuggled Migrants. In: Zapata-Barrero, R., Yalaz, E. (eds) Qualitative Research in European Migration Studies. IMISCOE Research Series. Springer, Cham. https://doi.org/10.1007/978-3-319-76861-8_15

 One aspect of the ethical concerns might be addressed if the text would briefly break down what types of QA texts are being referred to in each context, whether written, audio, video, images or multimedia, and in which contexts they may or may not be reliable as archival qualitative research sources for more effective collaboration between migration research and policy. For example, if “QAR facilitates an exploration of the values and reasons behind migration in the various cultures and can assist in explaining the different elements of migration of varying significance to the overall concept”, then written, audio, video, images or multimedia may be conceived and interpreted differently in different cultures. For example, in iconoclastic cultures or contexts with technological biases or access problems, written or audio texts may carry more weight and reliability than visual and multimedia texts. Pointing out how these elements can lead to different responses in meeting discussions would reinforce the results.

**Analysis (key findings)**

 The findings section would benefit from including more references to link some points with current literature. For instance: “Pose new questions to policy-decision makers and practitioners, by allowing them to ground on an in-depth knowledge.” From whom? The so-called ‘experts’? Are some of these experts migrants? Addressing the crucial issue of epistemic justice in this subsection would highlight the potential of QAR as a contextualised, interrelational, and reflexive methodology that can complement and interpret quantitative data, as this is one of the central insights of the open letter. 

 One quote per finding would give the reader an idea of the tone of the discussions at the meetings. Ideally, one quote from each leading group would be representative of the diversity of participants and voices: for example, policymakers or government, researchers/practitioners, civil society/non-governmental migrant organisations/migrants. In this way, the reader is encouraged to compare the opinions of entities/individuals who represent and shape migration research/policy from above and from below, fostering critical thinking and engagement with the different findings.

**Conclusions (recommendations)**

 The recommendations could serve as conclusions. Maybe the points could be better organised following a rationale: for instance, the need for contextualisation (understanding the time-space conjuncture and historical colonial continuums, points 1, 2, 4, 5), reflexivity (points 2, 3), interdisciplinarity (points 6, 8, and potentially 9), and critical action (7, 10, 11).

**Other comments:**

 To reinforce the mentioned aspect of using QAR to foster the agency of people who migrate, I recommend the reading:

- Reed, M.S., Merkle, B.G., Cook, E.J. *et al.* Reimagining the language of engagement in a post-stakeholder world. *Sustain Sci* **19**, 1481–1490 (2024). https://doi.org/10.1007/s11625-024-01496-4

As QAR is fundamentally about discourse and, therefore, the interpretation of any type of text, it is essential to be self-reflective about the language in use. Such self-reflexion can significantly influence the critical standpoint of the Open Letter.

Where applicable, are recommendations and next steps explained clearly for others to follow? (Please consider whether others in the research community would be able to implement guidelines or recommendations and/or constructively engage in the debate)

Yes

Does the article adequately reference differing views and opinions?

Partly

Are all factual statements correct, and are statements and arguments made adequately supported by citations?

Partly

Is the rationale for the Open Letter provided in sufficient detail? (Please consider whether existing challenges in the field are outlined clearly and whether the purpose of the letter is explained)

Yes

Is the Open Letter written in accessible language? (Please consider whether all subject-specific terms, concepts and abbreviations are explained)

Yes

Reviewer Expertise:

Forced migration, borders, film, participatory research, participatory filmmaking, self-representation

I confirm that I have read this submission and believe that I have an appropriate level of expertise to confirm that it is of an acceptable scientific standard, however I have significant reservations, as outlined above.

## References

[1] : Qualitative Archives and Biographical Research Methods. An Introduction to the FQS Special Issue. *https://www.researchgate.net/profile/Maria-Tamboukou/publication/274832353_Qualitative_Archives_and_Biographical_Research_Methods_An_Introduction_to_the_FQS_Special_Issue/links/552a50080cf29b22c9bf8874/Qualitative-Archives-and-Biographical-Research-Methods-An-Introduction-to-the-FQS-Special-Issue.pdf?origin* .2011;

[2] : Reimagining the language of engagement in a post-stakeholder world. *Sustainability Science* .2024;19(4) : 10.1007/s11625-024-01496-4 1481-1490 10.1007/s11625-024-01496-4
